# Development of an HPLC-DAD method for the simultaneous analysis of four xenoestrogens in water and its application for monitoring photocatalytic degradation

**DOI:** 10.1016/j.mex.2022.101789

**Published:** 2022-07-20

**Authors:** Khirbet López-Velázquez, Jorge L. Guzmán-Mar, T. Montalvo-Herrera, Sandra Y. Mendiola-Alvarez, Minerva Villanueva-Rodríguez

**Affiliations:** a Universidad Autónoma de Nuevo León (UANL), Facultad de Ciencias Químicas, Av. Universidad s/n, Ciudad Universitaria, San Nicolás de los Garza, Nuevo León, 66455, México; b Universidad de las Islas Baleares, Departamento de Química, Palma de Mallorca, 07122, España

**Keywords:** HPLC method, Xenoestrogens, Endocrine-disrupting compounds, Water pollution, HPLC-DAD, High-performance liquid chromatography with diode array detector, E2, 17β-estradiol, EE2, 17α-ethinylestradiol, BPA, Bisphenol A, 4TOP, 4-tert-octylphenol

## Abstract

A high-performance liquid chromatography with diode array detector (HPLC-DAD) method was developed and validated for the simultaneous quantification of 4 xenoestrogens in water for monitoring their photocatalytic degradation in synthetic water. The analytical parameters evaluated were linearity, limits of detection, and quantification (LODs and LOQs), selectivity, and accuracy, according to the US Food and Drug Administration (FDA) and Eurachem guidelines. The developed method shows good linearity (R^2^ > 0.995 for all compounds), and LODs ranged from 0.02 to 0.04 mg L^−1^, while LOQs ranged from 0.05 to 0.11 mg L^−1^. Moreover, accuracy expressed as recovery and precision were within the required limits. Therefore, the developed method was considered accurate, and reliable. In addition, it was successfully applied for monitoring a mixture of 4 xenoestrogens in water during the photocatalytic treatment.

• An HPLC-DAD method was developed to quantify 4 xenoestrogens in water simultaneously.
• The developed HPLC-DAD method shows excellent linearity, selectivity, and accuracy.
• A mixture of 4 xenoestrogens was reliably monitored during their photocatalytic degradation.

## Graphical abstract

**Figure:**
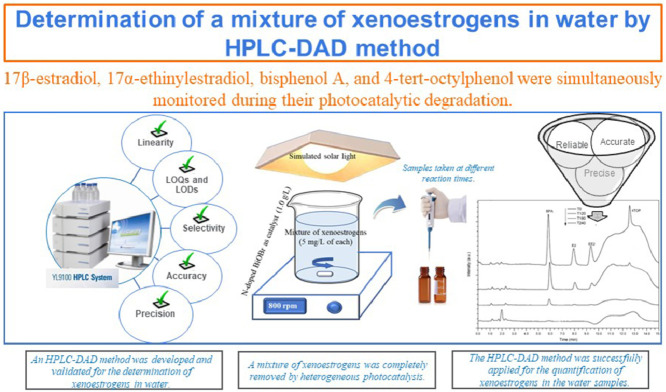

Specifications table

**Table:** 

Subject Area:	Environmental Science
More specific subject area:	*Analytical chemistry*
Method name:	*Simultaneous monitoring of four xenoestrogens in water by HPLC-DAD*
Name and reference of original method:	*Efficient photocatalytic removal of four endocrine-disrupting compounds using N-doped BiOBr catalyst under UV-Vis radiation. López-Velázquez et al., (2021). J. Env. Chem. Eng. 9(5), 106185.*10.1016/j.jece.2021.106185*.*
Resource availability:	*N.A.*

## Introduction

The occurrence of xenoestrogens as 17β-estradiol (E2), 17α-ethinylestradiol (EE2), bisphenol A (BPA), and 4-tert-octylphenol (4TOP) in water sources represents a relevant environmental problem due to their high potential to cause serious adverse effects on human health and aquatic life. The xenoestrogens may cause endocrine disruption in the aquatic organisms, and the chronic exposition to these micropollutants can lead to the possible collapse of population, intersex, and reproductive disorders in several species [1]. Therefore, developing and assessment of new technologies that efficiently remove this type of micropollutants from water are of great importance. In this sense, heterogeneous photocatalysis is an option to remove xenoestrogens from aqueous medium, emphasizing the development and application of active semiconductors under visible light and solar light. In our related study, N-doped BiOBr semiconductor was employed to completely remove a mixture of E2, EE2, BPA, and 4TOP in spiked samples under simulated solar light; for detailed information and an overview about these work, please refer to the full-length article [2]. The aim of this work was to develop and validate a quick and easy HPLC-DAD method for the reliable determination of a mixture of E2, EE2, BPA, and 4TOP in water for its application in the monitoring during the photocatalytic degradation, which represents a contribution to the development of new accessible methodologies for the analytical assessment of xenoestrogens in remediation assays.

## Method details

### Chemicals

17β-estradiol (≥98.0%), 17α-ethinylestradiol (≥98.0%), bisphenol A (≥99.0%), 4-tert-octylphenol (≥99.0%), and acetonitrile HPLC grade were purchased from Sigma-Aldrich (St. Louis, MO, USA). Water HPLC grade was acquired from Tedia Company (Fairfield, OH, USA). The main characteristics of the studied compounds are shown in Table 1.

**Table 1:** Characteristics of the studied xenoestrogens.

	Chemical formula	Molecular mass (g mol^−1^)	Log K_ow_	Solubility (mg L^−1^)	Use	MPC^a^ proposed by the European Parliament
E2	C_18_H_24_O_2_	272.38	4.0	28.0	Natural estrogen	0.001 µg L^−1^[3]
EE2	C_20_H_24_O_2_	296.40	3.6	11.3	Synthetic estrogen	0.035 ng L^−1^[4]
BPA	C_15_H_16_O_2_	228.29	3.3	120	Plasticizer	0.01 µg L^−1^[3]
4TOP	C_14_H_22_O	206.32	5.2	5.0	Surfactant	2 µg L^−1^[5]

a Maximum permissible concentration in surface water.

### Preparation of standard solutions and mobile phase

Stock solutions of E2, EE2, BPA, and 4TOP at 500 mg L^−1^ were prepared in acetonitrile, and work solutions containing the four analytes at 100 and 10 mg L^−1^ were prepared by dilution of stock solutions in distilled water. Six standard solutions (0.3, 0.6, 1.2, 2.5, 5, 10 mg L^−1^) were obtained by triplicate from the 10 mg L^−1^ working dissolution to construct the calibration curves. The stock solutions were stored at refrigeration (4 ± 0.5 °C), and work solutions were prepared as needed.

Water-acetonitrile mixture was used as mobile phase without pH change, and it was filtered through glass microfiber filters (0.22 µm, 47 mm, Whatman Inc. USA) and sonicated during 15 min for degassing (40 kHz, at room temperature).

### Instrumentation

**Fig. 1:**
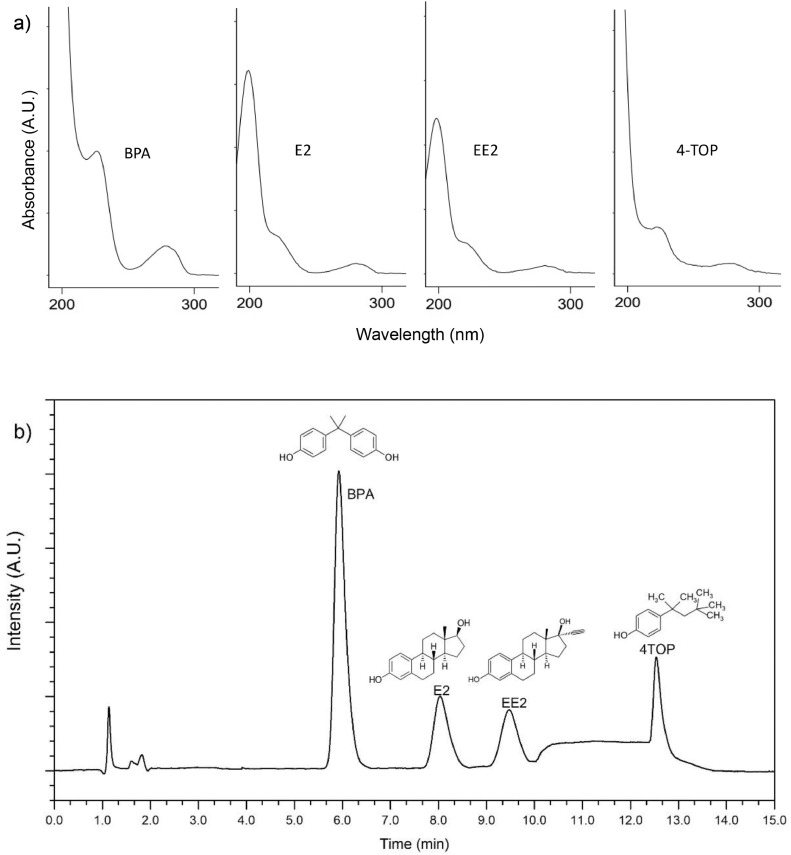
(a) Absorption spectra and (b) chromatogram of BPA, E2, EE2, and 4TOP mixture disolution (5 mg L^−1^ of each one).

The analysis of the xenoestrogens was carried out using a Young Lin (YL9100) system equipped with an autosampler, degasser, quaternary pump, and diode array detector. The separation of the analytes was carried out at room temperature using a Phenomenex Hyperclon C18 column as a stationary phase (250 mm × 4.6 mm, 5 µm). A mixture of acetonitrile-water at a constant flow rate (1.5 mL min^−1^) was used as a mobile phase; the proportion was changed by elution gradient according to Table 2 for the suitable separation of analogues E2 and EE2, and nonpolar 4TOP. The injection volume was 20 µL for standards and water samples, and the simultaneous detection of E2, EE2, BPA, and 4TOP was performed at a wavelength of 220 nm. Moreover, 15 min of run time was considered suitable for the analyses of these xenoestrogens. The spectra of each compound and the chromatogram are shown in Fig. 1.

**Table 2:** Gradient of acetonitrile-water used for elution of the xenoestrogens.

Time (min)	Acetonitrile (%)	Water (%)
0–7	40	60
8	75	25
10	75	25
11	40	60
15	40	60

### Samples preparation

The photocatalytic degradation of the compounds was carried out by triplicate in a Pyrex reactor using a synthetic solution prepared in distilled water containing the 4 xenoestrogens. The volume was 100 mL, and the initial concentration of the xenoestrogens was 5 mg L^−1^ each (pH 6.5). The loading mass of the catalysts employed was 1 g L^−1^ and their activation was carried out under solar simulated irradiation. For detailed information and discussion about the photocatalytic experiments, please refer to the related article [2].

During the photocatalytic experiments, 2 mL of samples were taken every 60 min for the quantification of the xenoestrogens. Before the HPLC-DAD analysis, the samples were centrifuged at 11,200 × g for 5 min (at room temperature) to separate the catalysts. Further, the supernatant (1 mL) was carefully transferred to amber vials and analyzed immediately. The filtration step did not use due to the adsorption of the less polar compounds in the filters.

### Method validation

The developed HPLC-DAD method was validated according to the US Food and Drug Administration (FDA) and Eurachem guidelines [6,7]; linearity, detection, and quantification limits (LODs and LOQs), selectivity, and accuracy were the analytical parameters evaluated.

The linearity of the method was evaluated through the calibration curves, which were constructed using six standard mixtures ranging from 0.3 to 10 mg L^−1^ of each compound. The standard mixtures were analyzed by triplicate and the peak areas versus concentration of the analytes were used to obtain the curves. The determination coefficients of the four calibration curves were >0.995, indicating good linearity and were considered suitable for quantification of the studied xenoestrogens in the water samples (Table 3).

**Table 3:** Data from the linear regression; LODs and LOQs values.

	Retention times (min)	Regression equation	(R^2^)	LOD (mg L^−1^)*n* = 3	LOQ (mg L^−1^)*n* = 3
BPA	5.9	*y* = 118.6x + 19.78	0.999	0.02	0.06
E2	8.0	*y* = 78.87x + 2.46	0.999	0.04	0.11
EE2	9.4	*y* = 59.10x – 17.82	0.995	0.02	0.06
4TOP	12.5	*y* = 35.97x – 1.06	0.999	0.02	0.05

Subsequently, data of the calibration curves were also employed for estimating of LODs and LOQs using the following equations [8]:

$$\text{LOD}=\left(3.3\times \sigma_{B}\right)/b;\;\text{LOQ}=\left(10\times \sigma_{B}\right)/b$$

where σ_B_ is the standard deviation on the blanks and b is the slope of the linear regression, the estimated values for LODs and LOQs are summarized in Table 3.

**Fig. 2:**
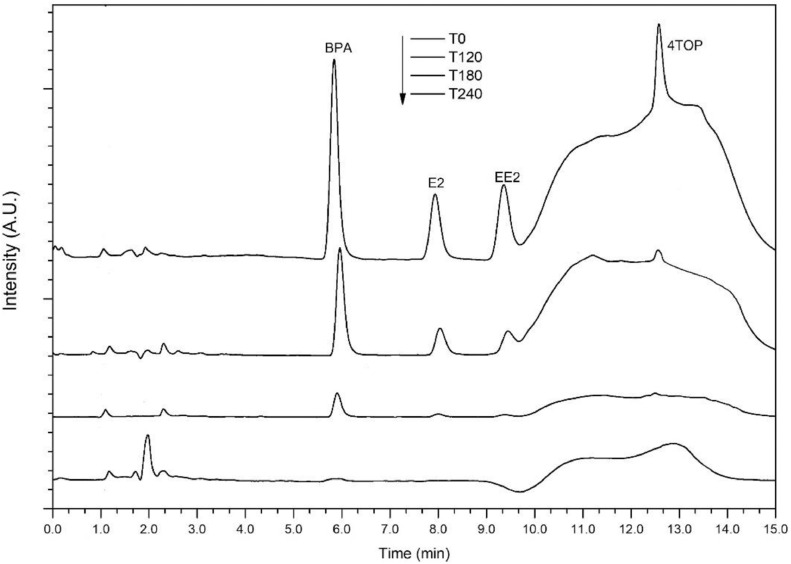
Evolution of chromatograms during photocatalytic degradation.

Method selectivity was assessed continuously by analyzing standard solutions (at 5 mg L^−1^, *n* = 12) and water samples taken from the reaction media (*n* = 12). The absence of coeluted peaks and signals from matrix interferences, as well as constant retention time, revealed the appropriate method selectivity as shown in Fig. 2.

In another way, the accuracy of the method expresses the closeness of agreement between the test result and an accepted reference value [9]. In this work the accuracy was estimated by determining the recovery (%) and the precision (expressed as relative standard deviation, %RSD). Both recovery and precision were established by analyzing three replicates of spiked samples at two levels of concentration: 1 and 10 mg L^−1^ of each compound. The acceptance criteria are the follows: for recovery, measured values should be within 15% of the level considered as the reference value, and for precision, the deviation of results (%RSD) should be lower than 15% [7]. Data estimated for recovery and precision parameters are shown in Table 4, and it is observed that all results are within the acceptance criteria of 15%, indicating that the developed method is accurate and reliable for the quantification of 4 xenoestrogens in the water samples.

**Table 4:** Results of the accuracy estimation.

	Recovery (%, *n* = 3)		Precision (%RSD) (*n* = 3)	
	1 mg L^−1^	10 mg L^−1^	1 mg L^−1^	10 mg L^−1^
BPA	101.2 ± 6.3	101.6 ± 3.8	5.1	3.1
E2	107.9 ± 5.1	99.6 ± 6.0	3.9	4.9
EE2	114.6 ± 5.6	100.2 ± 6.6	3.9	5.4
4TOP	108.1 ± 1.6	99.7 ± 4.6	1.2	3.8

### Method application

The validated method was applied for the monitoring of 4 xenoestrogens in water during their photocatalytic degradation. Fig. 2 shows four chromatograms corresponding to samples taken at different reaction times from the reaction media (0, 120, 180, and 240 min). A marked decrease of the compound´s concentrations by the effect of the photocatalytic treatment under simulated solar light was observed. At the end of the reaction (240 min), the levels of the compounds were <LOQ, and the absence of new peaks suggests the high efficiency of the photocatalytic process for the complete degradation of the contaminants, reaching the abatement of total organic carbon at levels of 53%. On the other hand, the absence of interference signals as well as coeluted peaks revealed the good selectivity of the method.

In literature, different HPLC methods have been developed and validated for the analysis of xenoestrogens in water. Among them, HPLC with electrochemical detection [10], HPLC with fluorescence detection [11,12], and HPLC-DAD [13], were applied for monitoring xenoestrogens during degradation processes in water [11,13]. However, it is worth noting that these methods employed the solid phase extraction (SPE) technique as a preconcentration step, with the drawback of increasing the costs and time of sample preparation for the analysis. On the contrary, using the HPLC-DAD method developed in this work, a proper performance for quick monitoring of 4 xenoestrogens in water (initial concentration of 5 mg L^−1^ each) during their photocatalytic degradation was achieved.

## Conclusion

The developed HPLC-DAD method shows good selectivity without interference signals and coeluted peaks, good linearity (R^2^ > 0.995 for each compound), and LODs ranging from 0.02 to 0.04 mg L^−1^, while LOQs ranged from 0.05 to 0.11 mg L^−1^. The accuracy assessed as recovery and precision was within the acceptance criteria required by FDA and Eurachem. Therefore, this method was appropriate for the quick and effective quantification of E2, EE2, BPA, and 4TOP during the photocatalytic experiments, saving time, costs, and effort, compared with other HPLC methods, without preconcentration step. The developed method represents a contribution to the environmental field since it may be used as a reference in further studies, and as a reliable analytical method for the determination of E2, EE2, BPA, and 4TOP in water samples taken from the degradation experiments, such as the advanced oxidation processes and bioremediation assays.
